# The Role of Boundary Spanning in Building Trust: A Place‐Based Study on Engaging Hardly Reached Groups in Community Healthcare Settings

**DOI:** 10.1111/1467-9566.13870

**Published:** 2024-12-23

**Authors:** Lara Bianchi, Mihaela Kelemen, Alysha Kate Shivji, Jonathan Tallant, Stephen Timmons

**Affiliations:** ^1^ Department of Entrepreneurship and Innovation Nottingham University Business School Nottingham UK; ^2^ Department of Strategy and Leadership EGADE Tecnologico de Monterrey Monterrey Mexico; ^3^ Department of Philosophy University of Nottingham Nottingham UK; ^4^ Centre for Health Innovation, Leadership & Learning Nottingham University Business School Nottingham UK

## Abstract

This paper investigates the impact of boundary spanning activities on building trust as a means of tackling health inequalities in hardly reached communities. Lack of trust has been identified as a barrier to engagement with healthcare services, resulting in poorer health outcomes. Engaging with hardly reached communities is challenging due to the social and symbolic boundaries prevalent in community healthcare settings. Drawing on empirical data from a recent year‐long collaborative research project with communities from seven economically deprived areas in the City of Nottingham, we identify two boundary spanning activities that facilitate the development of trust: communication across boundaries and intergroup relationship building. By cross fertilising sociological accounts on trust with insights derived from philosophy, the study finds that for hardly reached communities, trusting relevant individuals is more potent and widespread than the trust they have in healthcare institutions. By developing individual trust, hardly reached communities are more likely to consequently perceive the existence of institutional goodwill and competence. This counter‐intuitive finding invites us to regard trust as context specific and relational rather than as a binary choice between trusting individuals or institutions and to situate cross boundary activities focused on trust development within the power asymmetries in which they unfold.

## 1 Introduction

This paper explores potential ways to overcome barriers to engaging with ‘hardly reached’ groups (Sokol and Fisher 2016) to tackle health inequalities. Groups are hardly reached due to various intersecting demographics, as well as economic, environmental and place‐based factors including, but not limited to poverty, homelessness, race, disability, marital status and neurodivergency (Sokol and Fisher 2016). By employing Sokol and Fisher's (2016) ‘hardly reached’ label instead of labels such as ‘marginalised’, ‘vulnerable’, or ‘deprived’ (Munari et al. 2021; Wallace, Farmer and McCosker 2019), we aim to avoid ‘piggybacking on the distress of the poor’ (Heath 2007, 1301) and highlight the agency of those who lack economic, social or cultural capital and whose needs are not always understood by health services. This shift in terminology directs the responsibility towards the health service structures that fail to reach these groups rather than attributing blame for unequal access and poor health outcomes to the groups themselves (Orton et al. 2022; Wallace, Farmer and McCosker 2019).

Extensive research on health inequalities has revealed that individuals from hardly reached groups experience poorer health outcomes compared to the general population (Hui et al. 2020; Marmot 2020; Wallace, Farmer and McCosker 2019). While the UK government has received some acclaim for its research and policies aimed at tackling health inequalities, scholars point to the widening health inequalities gap in the UK despite unprecedented efforts to address disparities in recent years (Garthwaite and Bambra 2017; Kandt 2018; Smith and Anderson 2018).

Hardly reached groups face multiple intersecting challenges which increase their vulnerability. Symbolic challenges stem from dominant cultural norms which normalise stigma and prejudice against such groups (Hatzenbuehler and Link 2014; Østerud 2023; Silva, Durden and Hirsch 2023), while social challenges are escalated by existing healthcare organisational structures, policies and processes which are often ‘designed for an ‘ideal abstract patient’ […] with privileged economic and social assets’ (Halford et al. 2019, 16). Establishing trust with hardly reached groups is therefore widely recognised as an essential condition for engagement with healthcare services and research (Downey et al. 2015; George, Duran and Norris 2014; Wilkins 2018).

The article starts by outlining a sociologically informed understanding of trust which benefits from philosophical insights into goodwill and competence. It then highlights the potency and dangers of boundary spanning activities in building trust with hardly reached groups in community healthcare settings. This is followed by a discussion of our participatory methodology which puts the voices of hardly reached individuals at the heart of the project. Two types of boundary‐spanning activities emerge as central to the development of trust: communication across boundaries and intergroup relationship‐building. By situating such activities within the wider social relations and power asymmetries in which they unfold, the study finds that developing individual trust is essential to the perception of institutional goodwill and competence within community healthcare settings.

## 2 Trust and Health

Lack of trust is not only a significant barrier to engagement with healthcare services but also leads to ineffective approaches to healthcare interventions and poorer health outcomes for certain populations (G. Brown et al. 2014; Fischer et al. 2016; Webb Hooper 2019). Trust is a concept studied by a variety of disciplines. For our own part, we start our observations by drawing on insights from sociological and philosophical literature.

Many sociologists argue that trust is an essential requirement for the functioning of society, being a way of reducing what would otherwise be an unmanageable complexity. Luhmann (1979), for example, argues that in a complex modern society we are more likely to be trusting systems rather than individuals. Through our day‐to‐day experience of how complex systems work, we receive confirmation of our trust (or distrust) without having to understand the complex processes that underlie them (Luhmann 1990). Giddens (1990) supports this view, arguing that one of the consequences of modernity is a reduction in the amount of trust which is accomplished at the personal level and an increase in the trust placed in impersonal, often quite abstract systems or institutions.

We take a less committal view; that trust is *not* simply a binary opposition between the personal and the impersonal; trust is a dynamic, multi‐faceted and context‐dependent social phenomenon. Sometimes individuals trust another individual; at other times, individuals may trust institutions. Recent work (e.g. Donati and Tallant 2020) has shown that philosophical tools can be used to critique and refine the analyses of trust used in the business and sociological literature.

We follow that philosophical work on trust and allow for both trust in individuals and trust in groups, including institutions and systems (Pouryousefi and Tallant 2022; Tallant 2017). What unites philosophical accounts is that trust is driven by and conceptually connects, both goodwill and competence. In Noteboom's (1996, 990) words:

Trust may concern a partner’s ability to perform according to agreements (competence trust), or his intentions to do so (goodwill trust).

Crucially, whether we trust an individual is not a matter of whether they are *in fact* competent or *in fact* have goodwill towards us, but whether we believe them to be or to do so (Nooteboom 1996, 991). It is our perceptions and attitudes towards the relevant others that are central to the existence of trust.

As Jones (1996, 4) states:

I defend an account of trust according to which trust is an attitude of optimism that the goodwill and competence of another will extend to cover the domain of our interaction with her…

In the absence of goodwill or competence, of course, trust is likely to decline as we adjust our attitudes and beliefs accordingly (C. P. Long and Sitkin 2018; Nooteboom 1996). To return to whether trust is placed in individuals or systems, we adopt the theoretical position that the trust individuals may place in healthcare individuals (in their goodwill and competence) and the healthcare organisations (their goodwill and competence) can vary substantially, but that they can trust both. Just as we may believe that individuals have goodwill towards us, we may also believe the same (rightly or wrongly) of institutions themselves. For instance, Carbo‐Valverde et al. (2013) note that banking customers ascribe a range of characteristics to financial institutions, including those that underpin goodwill (sensitivity, commitment to the individual, etc.). We thus see no barrier to treating these goodwill accounts of trust as applicable to both individuals and institutions.

As demonstrated in the wider literature, the trust that we have in ‐for instance‐ banks, can differ from the level of trust that we have in bankers (e.g. Sapienza and Zingales 2012), though there is trust in both individuals and institutions. The same phenomenon may occur in health, where individual healthcare workers may be trusted very much (or very little) despite the fact that healthcare institutions are trusted very little (or very much).

The relationship between trust and health has received heightened interest owing to the Covid‐19 pandemic. Researchers have investigated the role of trust and distrust in vaccine uptake and the significance of patient trust in healthcare systems for preventing infections (Morales, Beltran and Morales 2022; Richmond et al. 2022). Prior to Covid‐19, existing research had established the link between distrust and poor health outcomes, low healthcare quality and increased healthcare costs (P. R. Brown and Calnan 2016; Fotaki 2014; Webb Hooper et al. 2019). When considering hardly reached populations, it has been argued that trust plays a crucial role in facilitating their engagement and in promoting access to appropriate and adequate healthcare services. However, trust is frequently found to be lacking (Cyril et al. 2015; Feldmann et al. 2007; Flanagan and Hancock 2010). Consequently, practitioners and public health researchers have emphasised the need for interventions that prioritise building trust to address health inequalities (Jaiswal 2019; Morales, Beltran and Morales 2022; P. R. Ward 2017). We argue that boundary spanning interventions can be effective at building trust in contexts where groups face symbolic and social boundaries in accessing healthcare services.

## 3 Boundary Spanning in Community Healthcare Settings

Understanding how to develop trust in the context of community healthcare services remains undertheorized and in need of study (P. R. Brown and Calnan 2016). This paper utilises the sociological concepts of boundaries and boundary spanning (Lamont and Molnár 2002; Wang, Piazza and Soule 2018) as a lens to investigate trust in the context of health inequalities. Boundaries can be both symbolic, that is conceptual distinctions and cultural traditions that can create or dissolve social differences, as well as social, defined as ‘objectified forms of social differences manifested in unequal access to and unequal distribution of resources (material and nonmaterial) and social opportunities’ (Lamont and Molnár 2002, 168).

Healthcare research has explored boundaries in various contexts including patient and clinical boundaries (Bishop and Waring 2019), boundaries within and between professions (Ernst 2020; Farchi, Dopson and Ferlie 2023; Islam et al. 2020), boundaries between policymakers and healthcare organisations (Currie, Finn and Martin 2007), boundaries between health officials and experts in COVID‐19 policymaking (Esmonde et al. 2024) and disciplinary boundaries (Liberati, Gorli and Scaratti 2016). These studies have highlighted that boundaries impede collaboration, hinder knowledge exchange and act as barriers to effective and inclusive healthcare.

Within the context of addressing health inequalities, Pedersen et al. (2017) have examined how public health professionals engage in different forms of boundary work to foster inter‐sectoral collaboration. Our paper focuses on boundary spanning, a type of boundary work comprising activities that ‘facilitate the flow of information, ideas, resources, or relationships across group boundaries’ (Wallace, Farmer and McCosker 2019, 367). In the context of community healthcare, boundary spanning activities aim to connect healthcare professionals and institutions with local communities in particular, ‘hardly reached’ ones (Evans and Scarbrough 2014; Fleming et al. 2023; Pedersen et al. 2017), to enable the latter to navigate more effectively the formal and informal healthcare systems and access care that aligns with their unique needs (Dowrick, Kelly and Feder 2020; Wallace, Farmer and McCosker 2019, Wallace et al. 2021).

A significant challenge to boundary spanning is posed by existing power asymmetries between groups (Dowrick, Kelly and Feder 2020; Fleming et al. 2023; McCartney et al. 2021; Snow, Tweedie and Pederson 2018). This challenge is particularly relevant for hardly reached groups (McCartney et al. 2021; Bradby et al. 2020) whose needs and aspirations are not always fully reflected by research underpinning health interventions. Power asymmetries are often framed as barriers to participation that can be minimised by addressing and accommodating the specific needs of hardly reached groups throughout the life course of healthcare research and interventions (e.g. Santana et al. 2018). In contrast to this approach where power is seen as something to be managed and minimised, we view power as a productive condition of possibilities that both enables and limits the social relations and therefore, the trust between diverse groups of people and institutions (Phillips, Frølunde and Christensen‐Strynø 2021). As McCartney et al. (2021, 24) suggest, ‘the opportunities and constraints offered by power relations at any point in time thus become central to understanding and identifying opportunities for reducing health and social inequalities’. Given that power dynamics can both undercut and enhance perceptions of goodwill and competence towards healthcare individuals and institutions, thus eroding or stimulating the development of trust, it is important to examine how specific boundary spanning activities work to develop perceptions of goodwill and competence towards individuals and institutions.

Some of the boundary spanning is carried out by institutional actors which in our research are represented by social prescribers (professional employees of the NHS who facilitate a non‐clinical approach to improving patients' health and well‐being), councillors (elected members of the local government) and community organisers (leaders of community groups, programmes, or community organisations, sometimes paid by local government). The latter are, however, members of the local community and their activities typically reflect the views and aspirations of the locals. Other boundary spanning activities are initiated at grassroots level by community volunteers (regular or frequent volunteers of community or other non‐profit organisations). Previous studies have examined the health benefits that such individual ‘connectors’ bring to hardly reached populations (Aoun et al. 2020; Wallace, Farmer and McCosker 2019). Expanding upon this literature, our research investigates the role of boundary spanning activities in generating trust and its implications for health inequalities.

## 4 Methods

Nottingham City Council commissioned this study to inform their Health and Wellbeing Strategy. A prior desk‐based investigation into the historic wider determinants of health inequalities in Nottingham identified a historical connection between trust, distrust and health in the city. In order to highlight community voices and in recognition of the difficulties in measuring trust, the current study generated data from a combination of 20 traditional interviews conducted by academics and 17 peer to peer interviews conducted by six peer interviewers. Several of the peer interviewers were participants in traditional interviews too (see Tables 1 and 2). We conducted traditional interviews with social prescribers, community organisers, community volunteers and one councillor, while the peer interviewees were asylum seekers, migrant workers, people with disabilities, certain religious communities' representatives and single mothers from the most economically deprived areas in Nottingham.

**TABLE 1:** Traditional interviews.

Interviewee	Sex	Length	Descriptor	Area
CV1	Male	1 h	Community volunteer with majority Black led churches Nottingham community; Nigerian migrant; student	Radford
CV2	Female	1 h	Community volunteer with mental health services for mothers; Nigerian migrant; student	By Jubilee Campus
CV3	Female	1 h	Community volunteer with Nottingham Muslim communities; Bangladeshi migrant	By Jubilee Campus
CV4	Female	1 h	Community volunteer with Nottingham community health volunteers organisation; student; Indian migrant	Hyson Green
C	Female	1.5 h	Nottingham councillor	Sherwood Rise
CM5	Female	1.5 h	Community member; student; Muslim women's community; Nottingham mental health services patient	Hyson Green
CM6	Female	1.5 h	Community member; mother; Muslim women community	Hyson Green
CM7	Female	1.5 h	Community member; suffers from chronic disability	Hyson Green
CO1	Female	1 h	Community organiser working with an organisation that supports dementia patients and their carers through singing and with an organisation that works with adults with learning disabilities	Sneinton
CO2	Male	1 h	Community organiser with resident's association of Nottingham community	New Basford
CO3	Male	1 h	Community organiser running organisation for Hong Kong migrants in Nottingham; Hong Kong migrant	Meadows
CM3	Female	1 h	Community member; Sri Lanka migrant; mother	Unknown
CO4	Male	1 h	Community organiser run local community organisation and food parcel delivery in Nottingham; Nigerian migrant	Radford
CV5	Male	1 h	Community volunteer in local food pantry; Nigerian migrant	Radford
D‐CV6	Male	1 h	Community volunteer in local food pantry; migrated from India (missionary parents)	Radford
P‐CO7	Female	1 h	Community organiser of organisations working with community gardens and social eating initiatives	Edwalton
Ms‐CO5	Female	1 h	Community organiser of social eating initiatives organiser and veteran advocate in Nottingham; academic	Sneinton
CO6	Female	1 h	Community organiser; member of Black women community organisations in Nottingham; works at local organisation working with local communities and the waterways in Nottingham; was previously a social prescriber	Clifton
SP1	Female	1.5 h	Social prescriber	Bestwood
SP2	Female	1.5 h	Social prescriber	Meadows

**TABLE 2:** Peer interviewers.

Peer interviewer	Peer interviewee	Sex	Length	Descriptor
SP1	SP1_1	Male	50 min	British; lives in Bulwell
	SP1_2	Female	50 min	British; lives in Bulwell
	SP1_3	Female	42 min	British; lives in Bulwell; mother
	SP1_4	Female	35 min	Jamaican migrant
CV3	CV3_1	Male	32 min	Student; lives in Clifton
	CV3_2	Male	32 min	Student; lives in Clifton
	CV3_3	Male	22 min	Student; Muslim
	CV3_4	Female	22 min	Teacher; migrant
	CV3_5	Male	22 min	Student; Sudanese migrant
CV2	CV2_1	Female	43 min	Midwifery student; former midwife; Ghanaian migrant
	CV2_2	Female	52 min	Student; Nigerian migrant
CM2	CM2_1	Female	21 min	British; mother
	CM2_2	Female	32 min	Chinese migrant
	CM2_3	Male	33 min	Black community member in Bulwell
CV9	CV9_1	Male	13 min	Student; Ugandan migrant
	CV9_2	Female	7 min	Student; British
	CV9_3	Female	7 min	Indian migrant; mother

We began our research with participatory creative workshops in the most economically deprived areas in Nottingham, namely: Nottingham City East; Nottingham City South; Bestwood and Sherwood; Bulwell and Top Valley; Clifton and Meadows; Bilborough, Basford, Beechdale, Aspley, Cinderhill, Hyson Green and Strelley (BACHS primary healthcare network) and Radford. The workshops were co‐designed with and facilitated by an award‐winning community theatre and involved 81 community members, community organisers/volunteers and social prescribers across all seven areas. They provided a forum for building trust with the local communities which allowed us to identify respondents for our interviews as well as locals who were willing to undertake research methods training in order to carry out peer to peer interviews. The data from the workshops does not form part of this paper.

Peer interviewing, ‘a participatory method that is used to access hard‐to‐reach populations and communities’ (Warr, Mann and Tacticos 2011, 338) relies on peer researchers who have trusting relationships within their communities and better access to the field compared to traditional researchers (Devotta et al. 2016; Elliott, Watson and Harries 2002). It has been argued that interviewees feel more comfortable sharing their experiences of trust and distrust with peer researchers who come from their own communities (Guta, Flicker and Roche 2013).

The peer interviewers were trained in basic interviewing techniques. The 3‐h training session held at the university included an overview of different types of interview questions and effective approaches to active listening. Participants engaged in role‐playing exercises and were given an interview guide along with a voice recorder. They were tasked with conducting up to three interviews over 3 months with members of their communities to learn about their experiences of trust and health. All peer interviewers participated in an ethics briefing to learn about the potential ethical issues involved. Peer interviewers were paid for participating in the training and for completing the interviews.

All interviews took a narrative approach to make space for participants' reflections on their experiences of trust as it relates to health. ‘Narrative interviews place the people being interviewed at the heart of the research study’ (Anderson and Kirkpatrick 2016, 631) and therefore were most appropriate for our data collection in that they prioritised the voices of community members. This type of interviewing has been used effectively to generate data regarding health and illness in prior studies (Mackenzie, Skivington and Fergie 2020). Our research prompts aimed to elicit narratives about who the respondents trusted with their health and wellbeing and why, perceived barriers to trusting individuals or institutions and possible ways of overcoming them. Interviewees were also asked to recount incidents when their trust had been betrayed in the context of health and how these made them feel.

The interviews were recorded, transcribed and analysed using thematic analysis (Braun and Clarke 2022). We read the transcripts individually to pinpoint recurrent themes (Creswell 2013). We then discussed and compared the themes and agreed on five main categories: boundaries, power, communication, relationship‐building and trust. The initial findings were presented to some participants to ascertain whether these categories resonated with their lived experiences. In the final phase, these categories were ‘thickened’ and given conceptual rigour by referring to the extant literature on health inequalities and trust.

The University of Nottingham Research Ethics Committee provided ethical approval for this research. By adopting a reflexive, inclusive and relational approach to our research, we were able to capture rich data about sensitive issues (Eisner 2008; Keifer‐Boyd 2011) and through triangulating data sources (Denzin 1978) we ensured both the coherence and rigour of our findings.

## 5 Findings

This section presents our findings from data analysis to identify two types of boundary spanning activities which played a crucial role in building the perception of goodwill and competence towards individuals and institutions: communication across boundaries and intergroup relationship‐building.

### 5.1 Communicating Across Boundaries

The importance of communication across boundaries was discussed in the interviews in terms of *translating for inclusivity* and *being listened to*. A significant number of our respondents see communication as vital to overcoming distrust and in establishing trust with hardly reached groups. One of the community volunteers said:

?One of the ways we can overcome the distrust is by communicating with our community. We try and give them the information in a way they can understand so they will trust it.

(CV1)

Community members and volunteers talked about the importance of inclusive communication and possible strategies for developing attitudes and perceptions of goodwill and competence towards relevant organisations via inclusive representation. In the words of a community organiser:

People have got to trust organisations. […] it's having people that look like yourselves, look like the communities that we say we want to help. So, if you haven't got somebody from a community within that organisation that's trying to deliver something, how are they going to trust and move forward?

(CO6)

Moreover, they argued that translating or adapting communications to suit the needs of various local communities is important to building trust. Translation activities are embraced by both social prescribers and community organisers. For example, social prescribers talked about how they employ accessible language that avoids medical jargon and stigmatising terms in order to reduce their patients' distrust in health and social services by enhancing their understanding of available services.

A number of community organisers who worked with physically or mentally disabled individuals similarly emphasised the importance of adapting the style and content of communication to be more inclusive in order to generate trust in the healthcare system.

We try and address all the different learning styles and all communication styles that people need… for instance, I do a newsletter for XXX… it’s always in massive font size… and pictures and images as well… because not everybody can read, but they can get information from the image… realising that everyone communicates differently, so trying to be as inclusive as possible… that helps them trust the system.

(CO1)

Community volunteers also highlighted the power of foreign language translation for building goodwill and confidence in the healthcare service.

Language is such an important thing. We can’t expect someone, who doesn't even speak English, to believe something they can’t understand. So, if he or she hears about existing services in their own language, you know there's a way… the right language moves you closer to convincing the person to trust the system.

(CV4)

However, there are challenges in finding good translators, as emphasised by one community organiser:

We have lost a lot of interpreters because of people leaving the country. […] But we have to keep trying [finding interpreters] and keep all formats available. The language thing is something that is coming up a lot [within the community], because when people don’t understand the language, they are not going to trust the system.

(CO1)

One of the community volunteers highlighted the fact that translation is not sufficiently embedded in the healthcare system and that it tends to be focused on producing leaflets in different languages rather than in having translators available for telephone appointments.

The council produced leaflets in 25 languages. Whether that’s useful or not, I don’t know, […] because as soon as you ring the number, you get an answer in English. If a service is accessed through telephone, can we be sure that the language the caller speaks is available? A lot of mental health support happens over the phone. […] So the block is at the very beginning even before you get to the health issue. In community organisations, there will be people who are conversant in that language ‐ so whoever is planning whatever health improvement programme, they need to allow them to facilitate.

(CV6)

The importance of listening to the concerns and the needs of hardly reached individuals and groups also emerged as a significant subtheme. One of the community organisers stressed the importance of listening:

Basically XXX, our local councillor is very effective because she listens to people, she responds promptly and proactively and she's very hard working and competent… I think probably it's partly down to her personality and her skillset but it's also for the fact that she understands our community… she is a known and active member of the community working to promote the health and well‐being of people locally.

(CO2)

There is however a widespread perception that ‘*local people feel shut up or not listened to, so they just don't know where to go*’ (CO3). Social prescribers said that their role has been created by the NHS in order for people to have someone listen to their concerns. They argued that constant listening to people's needs is a key ingredient to building trust.

Just for the fact that we've got the time to listen. That builds trust.

(SP1)

It usually doesn’t happen right away… if you keep listening attentively and actively… you can start to build that trust.

(SP2)

Community volunteers acknowledged the widely held perception that hardly reached groups do not feel listened to and the importance of acting on what is learnt through listening, as the quote below illustrates:

Nobody is there to hear them, right? So, we make ourselves available to hear them. We may not really be the professional psychiatry to render the help they need at the moment, but actively listening can help build trust and then we can direct them to the necessary service organisations that can help.

(CV2)

It is clear from the data that communication across boundaries has the potential to establish trust with hardly reached groups and that a lack of translation and active listening may hinder other well‐intentioned efforts. We argue that translation and active listening foster trust precisely because they can build perceptions of goodwill and competence towards both healthcare individuals and organisations.

### 5.2 Intergroup Relationship Building

The data reveals that the boundary spanning activities observed encompassed also a strong relational dimension. Multiple participants in the study emphasised the significance of establishing meaningful long‐term relationships as part of building trust across boundaries. When discussing boundary spanning as a process of relationship‐building, participants underscored its *temporal and embodied* nature. Arguably, trust building necessitates time and patience as well as face to face interactions. The social eating spaces pioneered by Nottingham City were regarded as a successful initiative that helped build cross cultural and cross community relationships.

In the words of a community organiser:

I think developing good nurturing relationships takes a lot of time… That builds an awful lot of trust… these social eating spaces where people come to eat together and be together are great… they get to know the volunteers, they get to know the person that's doing the cooking. So, all of that builds trust.

(CO5)

The power of food sharing and collective eating was highlighted by another community member.

One way to build trust would be eating together… different communities and cultures sharing food… there’s definitely one way to build trust and foster relationships.

(CM3)

Community organisers and social prescribers emphasised their collaborative partnerships, with the latter frequently recommending the social eating spaces to socially isolated patients as a means to develop social relationships and build trust in the system.

I think a lot of social prescribers are really pleased to know that there's like networks of these spaces across the city… And I think that goes hand in hand with social prescribers as having sort of tried and trusted places where they know that they can safely signpost people.

(CO5)

Oh gosh, they [social eating spaces] have a socially massive impact… they create trust… I have one client who is severely anxious and hadn’t been out of his house in years and so I prescribed him to social dining at XXX and now he feels confident… he’s made relationships… he’s more trusting of the world… he would have probably ended his life if we if we hadn't have referred him… That's what we go to work for every day… it can really save somebody's life… social dining, community cafes, even if it's a cup of tea, you know, it's a space that's created. For people to connect with others… it takes time, but it is so powerful.

(SP2)

Another community organiser also discussed the impact of relationships in fostering trust and the pivotal role of trust in engaging with minority groups:

Getting to the point where your help is accepted is a very different thing. They only help accept help from people they trust. And I think that's basically the really, really big thing… when it comes to trust, it comes with those relationships… Trust… particularly if you're from a minority group, relationships really matter.

(CO7)

There are numerous stories in the data about the number of weeks, months, or even years it takes to build relationships across various sociocultural or medically‐derived boundaries. These stories shed light on the significant investment of time required to cultivate meaningful connections. This is particularly evident in the accounts of community volunteers establishing relationships with dementia patients and social prescribers forging connections with asylum seekers. A community organiser reflected on the emotions brought about by the realisation that building trust is such a time‐consuming process:

I was like, Oh my God. Like, I was teared up. I thought bloody hell. It's taken him seven years of coming to these things before he feels comfortable… it just really was a really light bulb moment to me that that really people, they need time.

(CO5)

A social prescriber emphasised the need for repeat consultation before trust could be developed:

We're able to build a rapport with that person. And it might take several interventions or consultations or whatever you want to call it to actually gain that trust.

(SP2)

Community members criticised superficial approaches to relationship‐building in which professionals or organisations ‘helicopter in and don't take the time to really engage and get to know or work with the community… and then they don't understand why the community doesn't trust them’ (CM3). A community organiser in the study reflected on the importance of time to developing trust in communities, referring to community initiatives that support health and well‐being by organising activities along local waterways:

It takes time for people to build up that trust and I think that's what's quite successful with what we do. It's not just a one‐off session and they're gone. It's a number of weeks. You know, the minimum of four weeks up to about 12 weeks. And when people see that familiar face and you get to know them and you get to understand how you know how they work and how they think, I think that makes it a lot. Easier for people to continue and want to carry on and see each other once the programme is finished.

(CO6)

However, even when relationship‐building processes are given time to build, not following up can sometimes lead to losing trust.

There was never any follow‐up… I've never heard whether they've done this health and safety audit, or if they had, what actions were taken. As a result, you know, so I would say that actually, I lost trust in the school.

(CO2)

Again, given our theorising about trust this is unsurprising. From the perspective of the community member there is no goodwill or competence on display, with many community members highlighting the importance of face‐to‐face interactions and the need to be physically accessible to communities to nurture those trust‐generating bonds.

## 6 Discussion

Within the realm of health interventions aimed at addressing health inequalities by engaging with hardly reached groups, we recognise the pivotal role of trust in fostering meaningful engagement across boundaries. Consequently, strategies for cultivating trust in such contexts are paramount (Fotaki 2014; Jaiswal 2019; P. R. Ward 2017). In his investigation into the impact of medical mistrust, Jaiswal (2019, 192) concludes that it is time for ‘the health care system to begin generating trust among underserved communities… to take active steps to dismantle trust and build trust’. Participants in our study described communication and relationship‐building processes that traversed various socio‐cultural boundaries as resulting in perceptions of goodwill and competence towards health professionals such as social prescribers and the healthcare system more generally. Indeed, while Giddens (1990) argues that in modern times, trust in the individual is superseded by trust in systems and organisations, we found the opposite to be the case. Our findings suggest that for hardly reached groups and individuals, establishing trust with relevant individuals (community organisers, social prescribers, translators, etc.) is highly important, acting in many cases as a stepping stone towards perceiving healthcare institutions as having goodwill and competence and therefore worthy of their trust. Our study extends previous research on the relationship between trust and health (Brownlie, Green and Howson 2008) by illuminating the socially embedded nature of interpersonal and institutional trust in community healthcare settings beset by social and symbolic boundaries. The focus on hardly reached groups allows us to scrutinise the complex relationship between vulnerability and trust from the point of view of those who lack economic, social or cultural capital.

Prior research has proposed boundary spanners as ‘trust ambassadors’ and studied their capacity to develop trusting bonds across boundaries to facilitate coordination and exchange of information (Coleman and Stern 2018; Wallace, Farmer and McCosker 2019). We identified two boundary spanning activities that are influenced by and influence the power relations that underpin the healthcare system: communication across boundaries and intergroup relationship‐building.

Translating communications in a context‐sensitive and inclusive manner was associated with enhanced perceptions of goodwill and competence towards healthcare professionals and the healthcare system. This finding supports existing communications research on culturally sensitive translation as a foundation for building trust (Purnell 2018) and the view that information translated into ‘user‐friendly vernacular’ is more likely to result in trust from users (McNie 2013). However, we found that translation is not always sufficiently embedded in the healthcare system and this has a detrimental impact on hardly reached groups' attitude to the healthcare system. The significance of listening to the voices of hardly reached groups, whose perspectives and needs are often overlooked, also emerged as a crucial dimension of building trust (Dutta‐Bergman 2004; O'Reilly‐de Brún et al. 2016) with the added contribution that only by acting upon the knowledge gathered through active listening, one can reduce distrust.

We also identified two factors associated with building meaningful relationships: the time it takes to build them, especially for people whose voices are not always heard by relevant institutions and the embodied nature of the relationship‐building process. The social eating spaces and initiatives were a case in point. For many participants in the study, the face‐to‐face and repeated interactions that take place in these safe spaces facilitated the relationship‐building process across different cultures and communities, despite the fact that they are city council‐funded initiatives. Shaw et al. (2023) similarly show in their research, investigating the impact of telehealth relationships of care, that physical proximity is foundational to developing trust in healthcare settings. Consistent with the conceptual framework proposed by Reeves, McConnell and Phelan (2023), who consider social inclusion as fundamental to interpersonal relationships, we see boundary crossing activities that enable communication across differences as key to intergroup relationship‐building and to developing attitudes of trust in the healthcare system.

The research has limitations inherent to studies involving hardly reached groups: barriers such as limited access, cultural differences and an initial lack of trust were mediated by our preliminary participatory workshops. Further, while utilising the peer‐researcher methodology has advantages, it also comes with limitations concerning the inexperience of peer interviewers, lack of time and their own subjectivity (Lushey and Munro 2015). Despite these challenges, our study offers valuable insights into engagement with hardly reached communities, emphasising the importance of trust generated from a combination of boundary spanning processes that are influenced and in turn influence the power relations that underpin healthcare delivery.

During the analysis phase of the research, it became apparent that the mitigation of distrust may be of even greater importance than the enhancement of trust. Hardly reached communities may sometimes harbour deep‐rooted distrust towards healthcare organisations and professionals; this underscores the necessity to address this distrust and to clearly signal goodwill and competence. Although some of our empirical data does discuss harmful processes that result in distrust, such as poor communication and the capacity for effective communication across groups to lessen existing distrust, we did not focus on the mitigation of distrust but on the development of trust. Thus, the majority of the data from the study cannot support claims regarding distrust. We recognise the often‐neglected distinction between trust and distrust (Hawley 2014) with distrust being often inaccurately treated as simply the absence of trust. However, distrust is an active process and feeling which occurs when individuals believe an organisation ‘ought’ to perform a particular action, but it does not.

## 7 Conclusion

This study contributes to the expanding body of literature investigating trust in the context of healthcare (P. Brown et al. 2011; Fotaki 2014; Legido‐Quigley, McKee and Green 2014; Richmond et al. 2022; Topp et al. 2022; P. R. Ward 2017; P. Ward, Coffey and Meyer 2015). It is notable, however, that while there is this extensive sociological literature on trust in healthcare, it has not extended so far with hardly reached groups, which is perhaps surprising given that trust is especially problematic for them. Our study aimed to understand how boundary spanning activities help develop trust in both healthcare professionals and organisations by helping hardly reached individuals negotiate symbolic and social boundaries and develop attitudes and perceptions of goodwill and competence towards healthcare professionals and organisations.

This research makes both theoretical and practical contributions. Theoretically, we contribute to scholarship on trust by cross fertilising sociological accounts with insights derived from philosophy. We provide a more nuanced understanding of trust in so far as it is moving beyond binary oppositions (such as trust in systems vs. trust in people). Trust in the theoretical literature is highly abstract (such as Giddens or Luhmann) and needs to be contextualised, especially in hardly reached communities. By doing so, we have found something new and surprising by applying goodwill and competence accounts of trust from the philosophical literature on trust to sociology. This is not something which, to the best of our knowledge, has been used elsewhere. Given concerns about declining levels of trust (Schilke, Reimann and Cook 2021) especially in state agencies, our findings suggest ways trust in these agencies can be maintained and enhanced. Further, given that some existing literature on trust in health care focuses on trust in systems, professions and processes (e.g. Gilbert 1998), our findings are an important reminder of the importance of trust in individual people.

While sociologists argue that trust in systems and rules is more prevalent in modern societies than individual trust, our study finds that for hardly reached communities, trusting relevant individuals is more potent and widespread than the trust they have in healthcare institutions. By seeing trust as an attitude of goodwill and competence that can emerge from activities that aim to span and dismantle social and symbolic boundaries in the context of hardly reached groups, we contribute to the growing literature that analyses the dimension of trust in healthcare (Brownlie, Green and Howson 2008; Ozawa and Sripad 2013; Petersen and Wilkinson 2017; Richmond et al. 2022; Topp et al. 2022).

On a more pragmatic level, our research demonstrates the importance of considering (dis)trust when developing policies and initiatives to tackle health inequalities. Our findings signal the need to invest more substantially in boundary spanners and boundary spanning activities. By understanding power not as something to minimise or control but as intrinsic to all social relations and therefore integral to boundary spanning activities, this enables policy makers to accept that engaging with hardly reached communities is itself a power laden process that could either foster or diminish trust in the healthcare system. Creating opportunities for developing attitudes of goodwill and competence towards individuals and organisations should be at the heart of healthcare interventions targeted at hardly reached populations.

## Author Contributions

**Lara Bianchi:** conceptualization (supporting); formal analysis (lead); writing–review and editing (equal). **Mihaela Kelemen:** conceptualization (supporting); formal analysis (supporting); methodology (lead); supervision (supporting); writing–original draft (equal); writing–review and editing (lead). **Alysha Kate Shivji:** data curation (lead); formal analysis (supporting); investigation (lead); writing–original draft (equal); writing–review and editing (supporting). **Jonathan Tallant:** conceptualization (lead); funding acquisition (lead); supervision (lead); writing–review and editing (equal). **Stephen Timmons:** writing–review and editing (equal).

## Ethics Statement

Approval was obtained from the ethics committee of the University of Nottingham. The procedures used in this study adhere to the tenets of the Declaration of Helsinki.

## Consent

Informed consent was obtained from all individual participants included in the study.

## Conflicts of Interest

All authors certify that they have no affiliations with or involvement in any organisation or entity with any financial interest or non‐financial interest in the subject matter or materials discussed in this manuscript.

## Data Availability

The data that support the findings of this study are available on request from the corresponding author (Mihaela Kelemen). The data are not publicly available due to (restrictions e.g. participants were ensured transcripts would not be shared when they consented to participate).
